# Exploring administrative staff’s acceptance of generative AI in Chinese vocational colleges: A UTAUT-guided thematic study

**DOI:** 10.1371/journal.pone.0346003

**Published:** 2026-07-17

**Authors:** Yangzi Zhang, Yuanyuan Tian, Kenny S. L. Cheah, Wen Fen Beh

**Affiliations:** 1 Henan Institute of Economics and Trade, Zhengzhou, Henan, China; 2 Institute of Advanced Studies, Universiti Malaya, Kuala Lumpur, Malaysia; 3 Faculty of Education, Universiti Malaya, Kuala Lumpur, Malaysia; 4 Faculty of Creative Arts, Universiti Malaya, Kuala Lumpur, Malaysia; University of Dhaka, BANGLADESH

## Abstract

As Generative Artificial Intelligence (GenAI) technologies reshape institutional processes, higher vocational colleges are increasingly exploring their potential in administrative management. Guided by the Unified Theory of Acceptance and Use of Technology (UTAUT), this qualitative study examines how administrative personnel perceive and accept GenAI in vocational college administration. In-depth, semi-structured interviews were conducted with 16 administrative staff from three vocational colleges in Henan Province, China. Data were analyzed using ATLAS.ti-assisted thematic analysis through a hybrid deductive–inductive coding strategy. A deductive seed codebook was developed based on the four UTAUT constructs, while inductive coding captured emergent themes. To enhance analytical rigor, two coders independently analyzed five transcripts, achieving an inter-coder agreement of Cohen’s κ = 0.84. Thematic saturation was reached after the 13th interview, with no new themes emerging from the subsequent sessions. The findings largely corroborate the relevance of the four core UTAUT constructs, performance expectancy, effort expectancy, social influence, and facilitating conditions, in explaining GenAI acceptance among administrative staff. Participants recognized GenAI’s potential to improve operational efficiency, decision-making, service responsiveness, and workflow standardization. However, they also expressed concerns regarding technical complexity, resources limitations, policy ambiguity, data privacy, accountability, and job security. These findings suggest several GenAI-specific contextual factors, including governance readiness, perceived trustworthiness and controllability, institutional digital maturity, and ethical safeguards. The study provides an exploratory, context-specific application of UTAUT to vocational college administration and offers practical guidance for GenAI training, governance, and responsible implementation.

## Introduction

The rapid advancement of Artificial Intelligence (AI), particularly Generative AI (GenAI) tools such as ChatGPT, is reshaping higher education by transforming how information is processed, decisions are supported, and institutional workflows are organized [1,2]. Recent empirical studies on AI and ChatGPT acceptance in higher education suggest that adoption is shaped by perceived usefulness, ease of use, social influence, trust, ethical concerns, and institutional support [1–5]. Consequently, higher education institutions face increasing pressure to integrate intelligent technologies not only into pedagogy but also into administrative functions. From a technology-acceptance perspective, this shift raises critical questions regarding how users evaluate the usefulness, usability, legitimacy, and institutional support conditions of GenAI within specific professional contexts. In China, this transition is bolstered by national and provincial policy initiatives. For instance, the Ministry of Education’s Action Plan for Artificial Intelligence Innovation in Higher Education (2018) emphasized AI research and institutional innovation, while the Henan Provincial “AI+” Action Plan (2024–2026) promoted AI-enabled development in education and public governance [6,7]. While these policy developments provide a necessary institutional framework, they do not explain how specific groups within higher education perceive, evaluate, and adopt GenAI in their daily operations.

Higher vocational colleges play a strategic role in preparing a technically skilled workforce. To fulfill this mandate, their administrative systems must adapt to more intelligent, data-driven forms of institutional management. GenAI, with its capacity for automated content generation, personalized interaction, and data-supported analysis, offers significant benefits for administrative decision-making and workflow optimization. However, unlike conventional digital tools, GenAI produces probabilistic outputs and may process sensitive administrative information. These features make acceptance dependent not only on perceived efficiency but also on trust, controllability, verification practices, and governance readiness. This aligns with emerging research emphasizing AI transparency and ethical safeguards as pivotal factors in educational technology adoption [8,9]. Despite this, the organizational conditions shaping administrative staff’s acceptance of such tools remain insufficiently understood.

While GenAI’s application in pedagogy has been widely explored, its adoption in institutional administration, particularly in vocational settings, remains underexamined. Existing studies have focused primarily on student learning experiences, faculty instructional use, or broad digital leadership. Conversely, a growing body of international research has begun to examine AI adoption beyond the classroom, including managerial decision-making, administrative awareness, and institutional readiness for AI-enabled transformation [10–14]. These studies indicate that AI adoption in higher education is as much an organizational and governance issue as it is a pedagogical one. However, these international research studies are often situated outside the Chinese vocational college context and provide limited qualitative insight into everyday administrative acceptance [13,14]. Less attention has been paid to administrative personnel, who operate at the intersection of governance procedures, institutional data, and policy implementation.

As central figures in governance and resource management, administrative personnel serve as critical gatekeepers of AI implementation. Their acceptance of GenAI is shaped by individual attitudes, organizational support, policy clarity, and perceived risks such as data privacy and job security. As administrative use is closely tied to institutional accountability and service quality. The Unified Theory of Acceptance and Use of Technology (UTAUT) is therefore an appropriate framework for this study, because it captures the conditions under which administrative staff evaluate technology adoption: perceived task value, ease of use, organizational and social endorsement, and institutional support.

In a GenAI context, these constructs require nuanced interpreted in relation to uncertainty, autonomy, and trust. Performance expectancy involves whether autonomously generated outputs are reliable for formal decisions. Effort expectancy includes the additional cognitive burden of verifying probabilistic outputs. Social influence assesses whether leadership legitimizes GenAI use in formal capacities. Facilitating conditions encompass data-governance rules and policy clarity. Therefore, the research gap lies in how administrative staff evaluate the behavioral dimension, including perceived usefulness, usability, willingness, and confidence; the organizational dimension, including legitimacy, leadership support, governance boundaries, and accountability; and the technological dimension, including infrastructure, system compatibility, data security, and output controllability within vocational-college governance.

To address this gap, the present study explores how administrative staff in Chinese higher vocational colleges perceive and accept GenAI technologies. Grounded in the UTAUT [5], the study investigates influencing factors across the four core constructs: performance expectancy, effort expectancy, social influence, and facilitating conditions. Using a qualitative thematic approach, it provides context-specific evidence on the factors shaping GenAI acceptance and offers implications for responsible AI adoption in vocational college governance.

This study makes three primary contributions. Theoretically, it applies UTAUT to the underexamined context of administrative staff in Chinese higher vocational colleges, identifying several GenAI-specific factors such as policy ambiguity, governance readiness, perceived trustworthiness and controllability, institutional digital maturity, and ethical/job-security concerns. Methodologically, it combines UTAUT-guided qualitative inquiry with semi-structured interviews, ATLAS.ti-assisted hybrid deductive–inductive thematic analysis, inter-coder reliability checking, saturation assessment, and institutional context portraits. Practically, it provides role-relevant guidance for GenAI training, policy clarification, data governance, verification procedures, and responsible implementation in vocational college administration.

## Literature review

### Unified theory of acceptance and use of technology (UTAUT)

This study employs the UTAUT to examine GenAI acceptance among administrative staff in higher vocational institutions. The UTAUT model, developed by Venkatesh et al. [15], synthesizes the Technology Acceptance Model (TAM) and other foundational technology acceptance frameworks [16]. Recent research has extended UTAUT and UTAUT2 to AI and ChatGPT acceptance in higher education, confirming the relevance of performance expectancy, effort expectancy, social influence, and facilitating conditions in GenAI-related adoption contexts [1,3–5].

In the present study, UTAUT is used as a sensitizing conceptual framework rather than a purely descriptive classification scheme. It informed the development of the interview protocol, the construction of the initial coding framework, and the interpretation of how administrative staff evaluate GenAI in relation to their work tasks, organizational environment, and institutional support conditions.

The UTAUT model comprises four core constructs: performance expectancy, effort expectancy, social influence, and facilitating conditions. It also incorporates moderating variables such as gender, age, experience, and voluntariness to examine their influence on the core constructs [15]. This structure is particularly suitable for administrative contexts because it links individual perceptions of value and usability with the institutional conditions that shape behavior. In this study, the four constructs were operationalized in relation to administrative functions rather than treated as abstract categories.

A GenAI-specific adaptation is necessary because GenAI differs from conventional digital tools. It produces probabilistic rather than fully deterministic outputs, generates institutional text autonomously, and often processes sensitive information. These features introduce unique concerns regarding accuracy, transparency, data privacy, accountability, and human oversight. Consequently, GenAI acceptance depends not only on perceived usefulness but also on whether users consider GenAI outputs trustworthy, controllable, and verifiable [9]. In this study, trustworthiness and controllability were treated as GenAI-specific interpretive mechanisms that shaped how participants evaluated performance expectancy, effort expectancy, facilitating conditions, and cross-cutting ethical concerns.

In the context of GenAI, performance expectancy refers to the belief that GenAI can enhance management efficiency and decision-making quality. Recent AI and ChatGPT acceptance studies similarly identified perceived usefulness and expected performance improvement as central determinants of adoption intention [1,2,10]. In managerial and higher-education administrative contexts, perceived AI benefits and digital literacy have also been linked to willingness to adopt technology for work-related tasks [11,12]. For vocational-college administrators, performance expectancy was interpreted through concrete work functions, including document drafting, information processing, workflow coordination, decision support, and service responsiveness. Because GenAI outputs may be incomplete, biased, or contextually inaccurate, performance expectancy was also interpreted through perceived AI trustworthiness and the extent to which staff believed outputs could be checked and controlled before being used in institutional decisions.

Effort expectancy denotes the cognitive effort and operational complexity required to use GenAI tools. Studies on ChatGPT acceptance have shown that perceived ease of use, AI literacy, and understanding of system limitations influence users’ attitudes and behavioral intentions [2,3,8]. In administrative settings, effort-related concerns may be intensified when AI-assisted outputs are used in formal workflows that require accuracy, accountability, and institutional compliance [11]. Accordingly, effort expectancy in this study was linked not only to interface complexity, but also to staff members’ digital literacy, understanding of GenAI functions, prompt-use confidence, and perceived burden of verifying AI-generated outputs. The generative and probabilistic nature of GenAI therefore expands effort expectancy beyond operational ease to include the additional work of checking, validating, revising, and taking responsibility for AI-supported outputs.

Social influence reflects the impact of leaders, colleagues, institutional norms, and policy expectations on administrators’ technology adoption. In GenAI-related studies, social influence has been shown to affect behavioral intention toward ChatGPT use, particularly when users perceive peer practices, institutional endorsement, or information quality as supportive [3,5,17]. In vocational education, organizational culture and commitment also influence staff engagement with innovation [18]. In the administrative setting, social influence was examined through leadership endorsement, peer demonstration, departmental norms, organizational culture, and the perceived legitimacy of GenAI use within institutional governance. For GenAI use, social influence also included whether leaders and colleagues treated AI-generated outputs as acceptable, transparent, and professionally legitimate in administrative decision-making.

Facilitating conditions refer to institutional resources that support GenAI use, including technical training, infrastructure, approved access, and policy guidance. Recent studies on faculty GenAI use and professional AI training similarly emphasized the need for structured support, training opportunities, and clear institutional guidance to enable responsible adoption [10,19]. Ethical concerns and trust are also increasingly recognized as important factors in AI tool adoption [9]. For administrative staff, facilitating conditions were conceptualized broadly to include training systems, hardware and network infrastructure, approved-tool access, data-governance rules, procurement arrangements, privacy safeguards, and accountability procedures. These conditions are especially important because administrative GenAI use often involves institutional data, formal documents, and cross-departmental workflows. Thus, facilitating conditions were extended to include GenAI-specific governance supports, such as approved tool lists, rules for handling sensitive data, verification procedures for AI-generated content, and clear responsibility boundaries when AI outputs are used in institutional work.

Furthermore, the UTAUT model accounts for the moderating effects of gender, age, experience, and voluntariness [15]. In the GenAI context, prior AI exposure, digital literacy, and institutional experience may shape how users interpret usefulness, effort, and organizational support [4,5,8]. Therefore, incorporating these moderating variables descriptively helps provide a more comprehensive understanding of GenAI acceptance in institutional administration. Given the qualitative design of this study, these moderators were not tested statistically. Instead, they were used to support cross-case interpretation of how prior AI exposure, administrative experience, department type, and institutional digital maturity shaped participants’ accounts of GenAI acceptance.

### Acceptance and use of generative artificial intelligence among administrative staff in higher vocational colleges

Current research in the Chinese context primarily focuses on the application and impact of GenAI in teaching and skills development within higher vocational institutions, particularly in areas such as instructional innovation, talent cultivation pathways, and industry-education integration. Zeng [20] highlighted that ChatGPT’s reasoning, text generation, and dialogic capabilities have contributed to the digital transformation of vocational education. Wu and Guo [21] proposed a GenAI-driven instructional reform framework for vocational colleges, while Xu et al. [22] reviewed the influence of ChatGPT and GenAI on vocational talent development and skills training models. Overall, Chinese scholarship has examined GenAI as a tool for pedagogical innovation, vocational skills development, and talent cultivation, while giving limited attention to administrative staff as a distinct user group in institutional governance.

Globally, AI adoption in higher education is increasingly recognized as an organization and governance issue. Recent studies on AI and ChatGPT acceptance have examined perceived usefulness, ease of use, trust, ethical concerns, and institutional support among students and faculty [1–5,10]. Beyond pedagogy, research has also addressed AI-supported decision-making, administrative digital literacy, digital leadership, staff awareness, and organizational readiness [11–14]. Phakamach et al. [13] surveyed administrators from vocational institutions in Thailand to develop a digital leadership model. Jackson [14] examined university human resource administrators’ awareness of GenAI in higher education and found both positive attitudes toward workflow improvement and concerns about data privacy, ethics, and job security. These studies show that administrative AI adoption is becoming part of the broader international discourse on higher-education digital transformation. However, a majority of the available research remains concentrated in university, faculty, managerial, or broad leadership contexts, while qualitative evidence on administrative staff in Chinese vocational colleges is limited. These international studies offer useful evidence on administrative awareness and digital leadership, but they are generally located outside the Chinese vocational education context and often rely on survey-based designs rather than qualitative exploration of acceptance mechanisms.

As summarized in Table 1, existing literature remains fragmented across three dimensions: research context, participant group, and methodological approach. Chinese studies provide in-depth discussions on teaching and talent development, whereas international studies put forth broader evidence on AI attitudes and administrative awareness. However, little is known about how administrative staff in Chinese higher vocational colleges interpret, negotiate, and accept GenAI within everyday institutional work.

**Table 1:** Comparison of domestic and international studies on GenAI adoption in vocational and higher education contexts.

Research stream	Main focus	Methodological tendency	Remaining gap
Chinese vocational-education studies	GenAI in teaching reform, skills training, talent cultivation, and industry-education integration	Mostly conceptual, policy-oriented, or pedagogical discussion	Limited attention to administrative staff acceptance and institutional governance
International studies	Digital leadership, staff attitudes toward AI, administrative awareness, and professional concerns	More frequently survey-based or quantitative	Limited contextual fit with Chinese vocational colleges
Present study	Administrative staff acceptance of GenAI in Chinese higher vocational colleges	Qualitative, UTAUT-guided thematic analysis	Addresses administrative perceptions, acceptance factors, and institutional conditions in a Chinese vocational context

This table contrasts the research focus, methodological tendency, and remaining gap across China-focused vocational-education studies, international studies, and the present study.

Within the Chinese context, systematic research into how administrative personnel interpret and negotiate GenAI within everyday work remain scarce. Therefore, adopting a qualitative approach, particularly thematic analysis, from the perspective of administrative personnel, offers valuable insights into the underlying influencing factors of adoption, providing a roadmap for the administrative modernization of higher vocational institutions in China.

## Materials and methods

### Ethics statement

This study was approved by the Research Ethics Committee of Henan University of Economics and Trade (HIET.TNC2.HIETREC-890). Participants were provided with written study information and contact details, allowing sufficient time for review and inquiry prior to participation. Informed consent was obtained from all participants, who were explicitly notified of their right to withdraw at any time without penalty.

Participants were informed that study findings might be disseminated through academic journals, conferences, public engagement events, and social media. To ensure confidentiality, all interview data were anonymized during transcription and analysis. Participants were assigned codes (P01–P16), and institutional identifiers were generalized as “Colleges A–C.” Any potentially identifying details were removed or generalized during manuscript preparation to protect the anonymity of both individuals and their respective institutions.

### Research participants

This study focused on administrative staff from three representative higher vocational colleges in Henan Province, China. Henan was selected as the research site due to its status as a major educational hub, hosting 114 higher vocational institutions, the highest number in the country. The province has actively embraced digital transformation and AI integration, providing a pioneering context with highly referential samples for understanding GenAI adoption in vocational administration.

**Fig 1:**
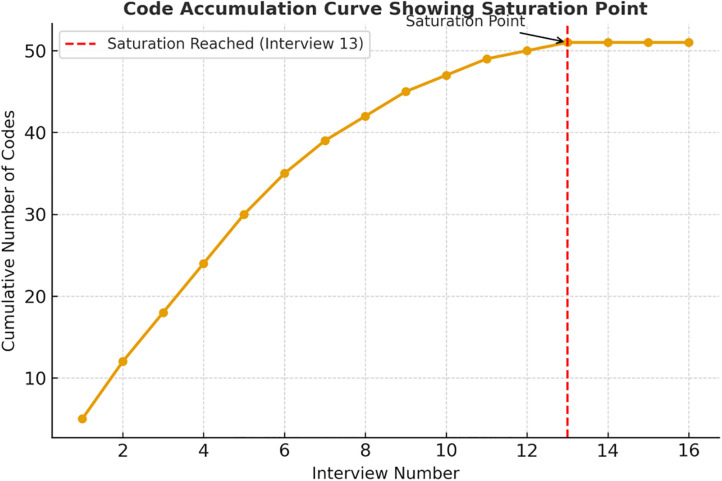
Code accumulation curve showing saturation point.

A purposive sampling strategy was employed to select 16 administrative staff members across three institutions. The sample size (n = 16) aligns with qualitative saturation principles [23], allowing for rich thematic exploration while ensuring analytical depth. Saturation was assessed during the coding process rather than assumed in advance. A saturation grid was maintained to track the emergence of new codes across successive interviews. As shown in Fig 1, new codes emerged until the 13th interview, after which the code accumulation curve plateaued. No new themes emerged from interviews 14–16, indicating that thematic saturation had been reached. Given the qualitative design, the purpose was not statistical generalization but analytical transferability through detailed contextual description. The study was conducted between September 2024 and February 2025.

Participants were drawn from eight functional departments: school leadership, human resources, academic affairs, student affairs, admissions and employment, finance, logistics, and college-level academic support units. Inclusion criteria prioritized individuals with substantial administrative experience and a strong understanding of institutional operations and technology implementation. This ensured the sample captured diverse perspectives across the key pillars of vocational college governance. Detailed participant characteristics, including job roles, years of experience, and prior AI exposure are provided in S1 Table.

### Institutional context portraits

To facilitate the assessment of transferability, contextual portraits are provided of the three participating colleges. These portraits were included to support thick description by clarifying the institutional conditions under which administrative staff evaluated GenAI adoption.

College A (a large-scale urban vocational college) enrolls approximately 18,000 students across engineering, business, and applied sciences programs. It maintains a relatively advanced digital infrastructure, with a provincial “smart campus” designation and an annual IT budget of roughly RMB 8 million. Its EdTech stack includes a cloud-based learning management system (LMS), integrated digital library resources, and pilot applications of AI-powered scheduling and plagiarism detection systems.

College B (a mid-sized regional college) serves around 9,500 students with strengths in manufacturing and logistics disciplines. Its digital infrastructure index is moderate, with partial smart campus coverage and an annual IT allocation of approximately RMB 4 million. Existing educational technologies include an internally developed student affairs system, limited use of AI-enabled language learning platforms, and routine deployment of office automation tools.

College C (a smaller practice-oriented college) enrolls about 6,200 students, specializing in service industries such as tourism, hospitality, and health care. It operates with constrained digital resources, allocating approximately RMB 2 million annually for information technology. Its infrastructure is basic, relying on standard office software, a local intranet-based academic affairs system, and initial trials of AI tools for online admissions and student counseling.

Together, these portraits illustrate variation in scale, resource endowment, and digital maturity across institutions, providing a context-rich basis for assessing the transferability of the findings to other vocational education settings. They also support cross-case interpretation of how institutional digital maturity shaped participants’ perceptions of facilitating conditions, policy clarity, and implementation readiness.

### Research method and questions

This study utilized one-on-one in-depth interviews guided by a semi-structured interview protocol titled “Perceptions and Influencing Factors of Generative AI Acceptance among Administrative Staff in Higher Vocational Institutions.” The interview protocol was validated through expert panel review and pilot testing with two administrators, ensuring content relevance and structural clarity [24].

Pilot feedback led to replacing technical jargon with accessible terms; for example, “algorithmic affordances” was revised to “system functions.” The sequencing of questions was also adjusted to distinguish more clearly between participants’ general perceptions of GenAI and the factors influencing their acceptance. As a result, the interview guide was shortened from 16 to 12 core questions, with clearer follow-up probes aligned with the four UTAUT constructs. The average duration of the pilot interviews was approximately 45 minutes, which informed the scheduling of the formal interviews [25]. Pilot data were used only to refine the interview protocol and were not included in the final thematic analysis. These refinements improved question clarity, reduced participants’ cognitive burden, and enhanced the conceptual alignment between the interview guide and the UTAUT framework. The final interview guide is provided verbatim in S1 File.

The interview framework is grounded in the UTAUT model, focusing on four key dimensions:

- Cognitive dimension: including time of initial exposure and information channels;
- Current usage dimension: including frequency and preferred tools;
- Acceptance dimension: encompassing willingness to use and attitudes toward AI integration in administrative tasks;
- Influencing factors: including perceived ease of use, anticipated improvements in efficiency and quality, and concerns regarding job stability.

Based on this framework, the study aims to address two core research questions:

1. What are administrative staff’s perceptions and attitudes toward the application of GenAI in institutional administration?
2. What are the key factors influencing their acceptance of GenAI?

### Reflexivity and positionality

The research team acknowledges the importance of reflexivity in ensuring rigor in qualitative inquiry. The first author is affiliated with a higher vocational college in Henan Province and thus occupies a partial “insider” position within the institutional context studied. This positionality afforded access to participants and facilitated trust-building but also carried the risk of gatekeeper bias and overly sympathetic interpretation of administrative perspectives [26]. Prior to data collection, the researchers held preliminary assumptions that generative AI could significantly enhance efficiency and decision-making in vocational administration. To mitigate the influence of these assumptions, reflexive journaling was maintained throughout the project, and coding decisions were discussed in peer debriefings with colleagues not directly involved in the research context [27]. The reflexive journal recorded interview impressions, emerging assumptions, coding uncertainties, and decisions made during theme refinement. Peer debriefing was used to challenge preliminary interpretations, especially where participants’ accounts could be interpreted as either individual hesitation or institutional constraint. Triangulation of codes across team members further reduced the risk of interpretive bias. These strategies were employed to bracket pre-understandings and ensure that emergent themes remained grounded in participants’ lived experiences rather than researchers’ expectations.

### Data collection and analysis

Interviews were conducted in Mandarin Chinese, the native language of all participants. The interviews were guided by a progressive questioning strategy and follow-up probing techniques, allowing participants to articulate their experiences, insights into influencing factors, and specific practices related to the use of GenAI in vocational college administration [28]. Recordings were transcribed verbatim in Chinese, with English translations provided only for manuscript reporting purposes to minimize meaning loss during analysis.

Selected quotations used in the manuscript were translated from Chinese into English by the first author and then checked by a bilingual researcher with experience in educational research. Any discrepancies in wording, terminology, or contextual interpretation were discussed until agreement was reached. During translation, contextual notes were retained where necessary to avoid loss of meaning, particularly for institution-specific expressions, administrative terminology, and culturally embedded descriptions of GenAI use. This process helped ensure that the English quotations accurately reflected the original Chinese interview data while remaining readable for an international audience.

For data analysis, the study employed ATLAS.ti 9.1-assisted thematic analysis. The analysis followed Braun and Clarke’s reflexive thematic analysis logic [29], adapted through a hybrid deductive–inductive coding strategy. It was not designed as grounded theory, because the aim was not to generate a new formal theory, nor was it treated as a purely deductive framework analysis, because inductive codes were allowed to emerge from the interview data. Rooted in interpretive phenomenology, thematic analysis focuses on identifying key themes and latent patterns within qualitative data, providing an in-depth portrayal of participants’ lived experiences and behavioral tendencies. The process followed six systematic steps [30,31]:

- Familiarization: intensive reading and annotation of interview transcripts;
- Coding: segmenting text and tagging meaningful information;
- Theme development: clustering codes and generating initial themes;
- Theme review: evaluating thematic validity and coherence;
- Theme definition: articulating theme characteristics and illustrative data;
- Narrative interpretation: situating themes within the research context, analyzing underlying meanings, comparing with previous studies, and extracting theoretical and practical implications.

A UTAUT-guided hybrid deductive–inductive coding strategy was employed to analyze the interview data. The first author led transcript familiarization, initial coding, and theme development, while a second independent coder with qualitative expertise analyzed a randomly selected subset of transcripts for reliability verification.

A deductive seed codebook was initially constructed based on the four core UTAUT constructs: performance expectancy, effort expectancy, social influence, and facilitating conditions. Each construct was defined with specific inclusion/exclusion rules and exemplar indicators. During the open coding phase, inductive codes were introduced to capture GenAI-specific emergent tehems that fell outside the initial UTAUT categories such as ethical/job-security concerns, policy ambiguity, data privacy, accountability, and trustworthiness of AI-generated outputs. The UTAUT moderating variables, including age, gender, experience, and voluntariness, were recorded descriptively in the participant matrix but were not utilized as analytic codes. No UTAUT constructs were discarded during the analysis. The finalized codebook, linking each UTAUT dimension to its thematic codes, indicators, and coding origin, is provided in S2 Table.

To ensure coding reliability, two independent coders analyzed five randomly selected transcripts, representing approximately 31% of the total dataset. Coding was compared at the thematic level using Cohen’s kappa (κ) statistic. The inter-coder agreement was calculated as κ = 0.84, exceeding the recommended threshold of 0.80 for substantial agreement [32]. Disagreements were resolved on a case-by-case basis through discussion and refinement of code definitions, with final coding decisions reached via consensus.

To enhance research transparency, a comprehensive audit trail was maintained throughout the analysis. Table 2 illustrates the theme derivation process, presenting exemplar quotes, associated initial codes, clustered candidate themes, final themes, and frequency counts. The audit trail documented changes to the codebook, reasons for merging or separating codes, consensus decisions between coders, and the movement from initial codes to candidate and final themes. Together, the audit trail, saturation grid, finalized codebook, cross-coder comparison, reflexive journal, peer debriefing, anonymization procedures, and translation verification process were used to strengthen the credibility, dependability, confirmability, and transferability of the qualitative analysis.

**Table 2:** Audit trail of theme derivation.

Exemplar Quote (Participant)	Initial Code	Candidate Theme	Final Theme	Frequency (n)
“GenAI helps me finish documents much faster, and the quality looks more professional.” (P04)	Improved document efficiency	Efficiency gains	Performance Expectancy	11
“At first, the system was confusing, with too many functions and jargon.” (P07)	Complex interface	Learning burden	Effort Expectancy	9
“When our director encouraged us to try AI tools, I felt more confident using them.” (P10)	Leadership encouragement	Institutional endorsement	Social influence	8
“Without proper training and hardware, it’s hard to use these tools effectively.” (P02)	Lack of resources	Resource limitations	Facilitating conditions	10
“I worry AI might take over some of our jobs in the future.” (P09)	Job security concern	Ethical/structural risks	Perceptions & Attitudes	7

This table illustrates the link between exemplar quotes, initial codes, candidate themes, final themes, and frequency counts.

### Trustworthiness

To ensure the quality and integrity of the findings, the study followed the four-dimensional criteria for trustworthiness in qualitative research.

First, credibility was supported through peer debriefing, cross-coder comparison, and consensus discussion. The first author and a second independent coder compared coding decisions for five randomly selected transcripts, and disagreements were resolved through discussion before the final codebook was applied to the remaining transcripts.

Second, dependability was enhanced through an audit trail that documented all modifications to the codebook, the rationale for merging or separating codes, and the conceptual progression from initial codes to candidate and final themes.

Third, confirmability was established through the use of reflexive memos, which recorded researcher impressions, coding uncertainties, and potential biases during the analytic process to ensure findings remained grounded in the data.

Fourth, transferability was strengthened through thick description, including institutional portraits of Colleges A–C, participant profiles, and exemplar quotations linked to each theme.

Fifth, triangulation was conducted across multiple dimensions, including coder perspectives, participant roles, institutional contexts, and data segments, to verify the consistency of emergent themes across departments and colleges.

Participants were invited to review summary themes, and their feedback was considered during final theme refinement. This limitation is acknowledged in the ‘Limitations and Future Research’ section. However, the use of verbatim Mandarin transcripts, bilingual quotation checking, audit trail documentation, and cross-coder comparison helped reduce the risk of misinterpretation. In addition, the Consolidated Criteria for Reporting Qualitative Research (COREQ) checklist was used as a reporting guide to improve transparency in describing sampling, interview procedures, coder roles, reflexivity, coding, and analysis.

## Results

Prior to presenting the thematic results, it is important to note that data analysis confirmed the achievement of thematic saturation. As illustrated in Fig 1, new codes continued to emerge until the 13th interview, after which the code accumulation curve plateaued. No novel themes were identified in the final three interviews, supporting the adequacy of the sample size (n = 16) for in-depth thematic exploration. Furthermore, inter-coder reliability checks indicated high consistency between coders (κ = 0.84), validating the robustness of the coding framework and the subsequent findings.

Overall, the results corroborate the relevance of the four core UTAUT constructs, performance expectancy, effort expectancy, social influence, and facilitating conditions, in explaining GenAI acceptance among administrative staff. Simultaneously, several GenAI-specific factors emerged inductively, including policy ambiguity, perceived AI trustworthiness and controllability, ethical and job-security concerns, and institutional digital maturity. Rather than replacing the four UTAUT constructs, these emergent factors nuanced how participants interpreted usefulness, ease of use, organizational legitimacy, and institutional support in vocational-college administration.

### Cross-case analysis

The three-college design facilitated a cross-case comparison based on institutional digital maturity, department type, and prior AI exposure. Participants from College A, which had stronger digital infrastructure and prior smart-campus experience, tended to emphasize performance gains, workflow integration, and the possibility of embedding GenAI into routine administrative processes. In contrast, participants from College B, with moderate digital infrastructure, more often described partial readiness: they recognized the potential of GenAI but stressed the need for clearer training, technical support, and departmental coordination. Participants from College C, where digital resources were more constrained, placed greater emphasis on basic infrastructure, equitable access, approved tools, and practical support before large-scale adoption could occur.

Differences were also observed across administrative roles. Finance and human-resources staff tended to emphasize data accuracy, privacy, compliance, and accountability when discussing GenAI use. Student affairs and admissions-related staff focused more on service responsiveness, communication efficiency, and personalized support, while also raising concerns about student data protection. Academic affairs and academic support participants frequently discussed document processing, information retrieval, scheduling, and workflow standardization. Logistics staff were more likely to connect GenAI acceptance to hardware, network conditions, and the practical feasibility of using AI tools in daily operations.

Prior exposure to AI tools further shaped participants’ interpretations of acceptance. Staff with higher prior exposure appeared more confident in experimenting with prompts, evaluating outputs, and identifying practical use cases. Those with moderate exposure often recognized potential benefits but requested structured training and examples from comparable departments. Participants with low or no prior exposure more frequently described unfamiliar terminology, uncertainty about correct use, fear of mistakes, and stronger dependence on institutional guidance. These cross-case patterns suggest that GenAI acceptance was not uniform across the sample, but varied according to institutional capacity, departmental responsibility, and individual experience with AI tools.

### Perceptions and attitudes toward generative AI among administrative staff in higher vocational colleges

The findings reveal a diverse and multilayered structure of attitudes among administrative personnel regarding GenAI implementation. While most respondents expressed an openness to the technology and its potential to streamline workflows, this optimism was tempered by psychological and practical barriers.

On one hand, technical knowledge gaps hinder independent operation in the short term. On the other hand, concerns regarding data security, ethical compliance, and job displacement contribute to diminished confidence in long-term adoption. Ethical concerns, especially data privacy, accountability for AI-generated outputs, and job security, emerged as cross-cutting factors. These indicate that acceptance is not solely a function of efficiency but depends on whether staff perceive GenAI outputs as trustworthy, controllable, and institutionally appropriate for formal administrative use.

**Fig 2:**
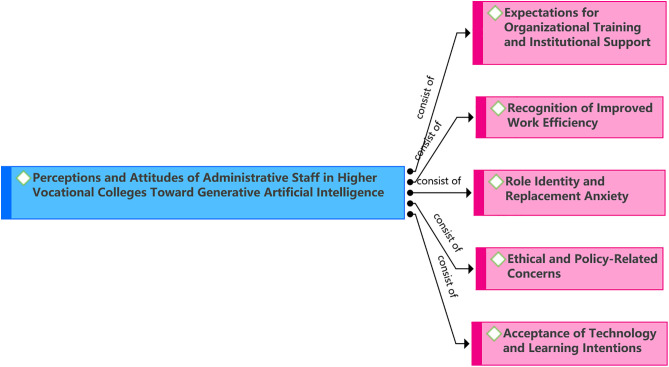
Perceptions and attitudes of administrative staff in higher vocational colleges toward generative artificial intelligence.

Participants consistently identified organization support as a prerequisite for adoption. They argued that structured training programs, clear regulatory frameworks, and institutional safeguards are essential for the effective use of GenAI. Successful implementation and sustainable development of GenAI in vocational college administration was seen as dependent on the maturity of external conditions, the guidance of organizational culture, and the continuous provision of technical resources. Consequently, findings suggest that institutional efforts should prioritize improving technological accessibility, enhancing training mechanisms, and refining policy support to build a sound institutional and practical foundation for the broader application of generative AI. Fig 2 presents the results of the thematic analysis.

### Factors influencing the acceptance of generative AI by administrative staff in higher vocational colleges

#### Performance expectancy.

The findings indicate that participants hold a predominantly positive outlook regarding the potential of GenAI to enhance professional performance. Respondents strongly recognized GenAI’s utility in improving administrative efficiency, optimizing decision support, enhancing service responsiveness, alleviating workload pressure, and promoting procedural standardization and transparency.

Many participants reported that GenAI tools could streamline routine administrative tasks, such as document drafting, official correspondence processing, and information retrieval, thereby increasing both the speed and quality of task execution. Additionally, the analytical and predictive capabilities of GenAI provide robust support for evidence-based and data-informed decision-making among managers.

Several respondents highlighted GenAI’s contribution to improving service quality and responsiveness, particularly through features such as real-time feedback, intelligent recommendations, and personalized services. These functions enhance the interactivity and user satisfaction associated with administrative operations.

**Fig 3:**
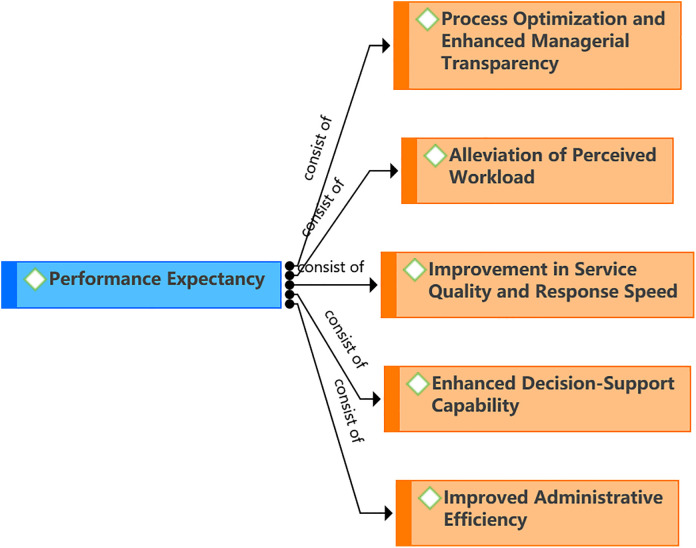
Performance expectancy as a factor influencing the acceptance of generative AI among administrative staff in higher vocational colleges.

Moreover, administrators acknowledged that GenAI reduces their cognitive and operational burden, allowing for a shift in focus toward high-value strategic responsibilities. Beyond individual task support, participants noted that GenAI advances the standardization and visualization of management processes, increasing the transparency and auditability of administrative practices. Fig 3 presents the thematic analysis results for the performance expectancy dimension.

#### Effort expectancy.

Within the dimension of effort expectancy, findings reveal that the perceived cognitive load and operational complexity associated with the use of GenAI significantly influence administrative staff’s willingness to adopt the technology. While interest remains high, participants identified several implementation barriers, specifically the time and energy required during the initial learning phase, as primary concerns.

The complexity of user interfaces and the intuitiveness of system interaction directly impact user experience. Administrators indicated that tools featuring clear, user-friendly design and human-centered interaction logic were more likely to foster ease of use and confidence. Perceptions of technological complexity varied among respondents. Some participants expressed feeling discouraged by specialized terminology and abstract algorithms, viewing them as high barriers to entry. These responses suggest that effort expectancy is shaped not only by system design but also by uneven digital literacy. Participants with higher prior exposure to AI tools were more confident in experimenting with prompts and interpreting outputs, whereas those with limited exposure more often emphasized unfamiliar terminology, uncertainty about correct operation, and dependence on step-by-step guidance.

Additionally, participants frequently emphasized the importance of error tolerance and the cost of trial-and-error when using AI tools. Concerns were raised regarding the potential risks of operational errors and accountability issues arising from misuse, making the presence of robust fail-safe mechanisms and recovery functions critical for sustained usage. For administrative staff, trial-and-error was not perceived as a purely technical learning process. Because their work often involves institutional documents, student information, finance-related procedures, or compliance-sensitive tasks, errors in AI-assisted outputs could create accountability risks. This made verification burden, responsibility boundaries, and the availability of safe experimentation spaces important components of effort expectancy.

**Fig 4:**
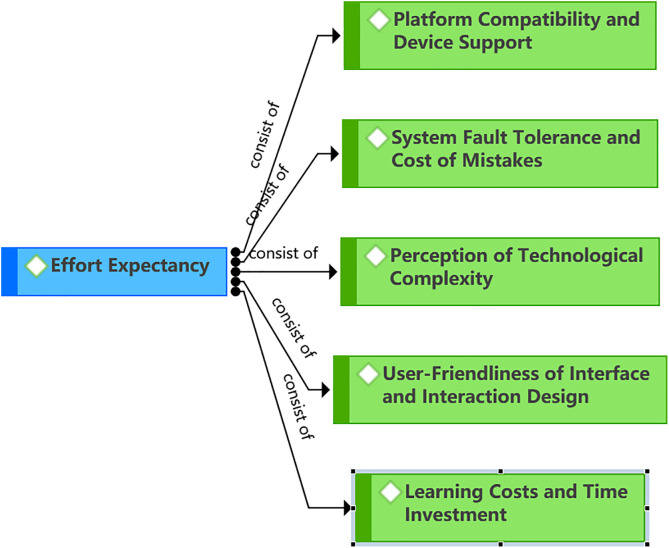
Effort expectancy as a factor influencing the acceptance of generative AI among administrative staff in higher vocational colleges.

Platform compatibility and hardware support were frequently cited as extrinsic effort factors. Participants noted that insufficient office equipment or overly complex access paths negatively impacted their motivation to use the technology. Motivation was higher when GenAI could be integrated into familiar workflows, such as document drafting, information retrieval, student affairs processing, or internal communication. In contrast, fragmented platforms, unclear access procedures, and limited institutional guidance increased the perceived burden of adoption. Organizational culture further influenced this process: staff in more innovation-oriented environments described greater willingness to experiment, whereas those in more risk-averse settings tended to approach GenAI use more cautiously. Fig 4 presents the thematic analysis results for the effort expectancy dimension.

#### Social influence.

Under the dimension of social influence, the study reveals that administrative staff in higher vocational institutions are significantly influenced by their organizational environment and interpersonal networks when adopting GenAI. This influence manifests through three primary channels.

The attitudes and behaviors of senior leaders are perceived as primary drivers of adoption. When institutional leaders actively promote AI tools and demonstrate their use in their own professional practice, it substantively enhances subordinates’ confidence and willingness to engage with the technology.

Peer dynamics play a critical role in the diffusion of GenAI. Participants noted that observing colleagues successfully use GenAI tools fosters a “social modelling” effect. This leads to conformity-based learning, where the sharing of experiences among peers creates a supportive micro-environment for technology uptake.

The foundational culture of the institution dictates the pace of adoption. Environments that advocate for innovation, tolerate trial-and-error, and encourage experimentation provide administrative personnel with a sense of psychological security. Conversely, a risk-averse institutional stance results in hesitation and resistance among employees.

**Fig 5:**
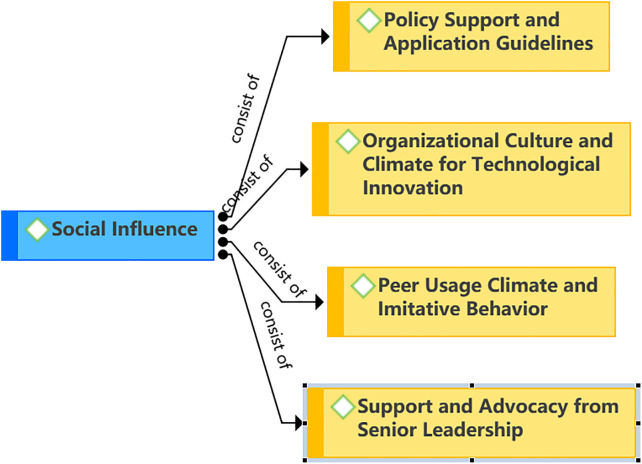
Social influence as a factor influencing the acceptance of generative AI among administrative staff in higher vocational colleges.

Moreover, the presence of explicit institutional guidelines serves as a critical social cue for adoption. When schools or relevant departments issue operational manuals and clear frameworks on GenAI usage, especially in regards to data security and regulatory compliance, administrators are more likely to trust and engage with the technology in practice. Fig 5 presents the thematic analysis results for the social influence dimension.

#### Facilitating conditions.

Within the dimension of facilitating conditions, the findings indicate that the availability of external resources and support systems significantly influences the decision of administrative staff to adopt GenAI. These conditions are perceived through four primary factors.

Structured training and capacity-building initiatives are viewed as essential prerequisites. Participants noted that continuous learning resources would substantially reduce barriers to adoption, enhancing their sense of control and operational confidence.

The adequacy of hardware, software, and network infrastructure directly affects the feasibility of GenAI utilization. Limitations in existing office equipment or restricted network accesswere found to impair platform performance and diminish user experience. Furthermore, institutional digital maturity emerged as a contextual moderator of facilitating conditions. Staff from better-resourced colleges tended to discuss workflow integration and advanced use scenarios, whereas those from resource-constrained settings emphasized basic access, equipment limitations, training availability, and policy guidance.

**Fig 6:**
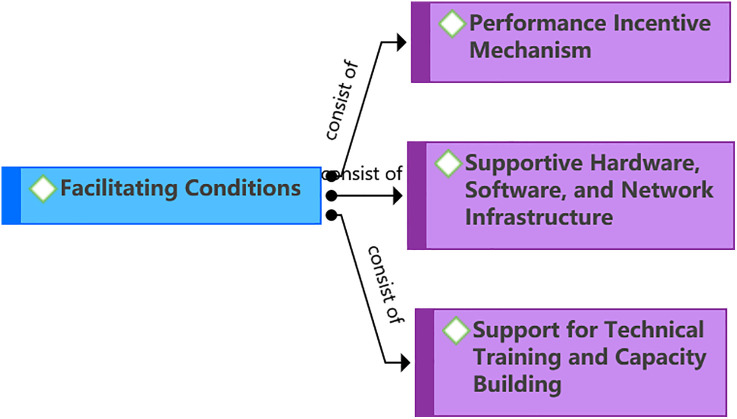
Facilitating conditions as a factor influencing the acceptance of generative AI among administrative staff in higher vocational colleges.

Incentive mechanisms emerged as a potential motivator for AI adoption. Participants generally agreed that incorporating AI utilization into performance evaluations, professional appraisals, or award systems would encourage staff to proactively invest in mastering these tools. Beyond technical resources, participants emphasized the need for governance readiness. This includes clear directives on approved tools, data protection protocols, output verification procedures, and accountability frameworks. These findings suggest that facilitating conditions for GenAI adoption extend beyond infrastructure and training to include policy clarity and institutional safeguards. Fig 6 presents the thematic analysis results for the facilitating conditions dimension.

To clarify the organizational and policy artifacts underlying facilitating conditions, Table 3 maps the main institutional supports and governance mechanisms raised by participants to specific adoption barriers and actionable institutional responses.

**Table 3:** Organizational and policy artifacts related to facilitating conditions for GenAI adoption in vocational-college administration.

Organizational/policy artifact	Related facilitating condition	Main issue raised by participants	Actionable institutional response
Data-governance policies	Policy clarity and responsible use	Participants were uncertain about whether GenAI could be used with institutional, student, or personnel data.	Develop clear data-governance policies specifying what types of data may or may not be processed through GenAI tools.
Approved-tool lists	Safe and legitimate access	Staff reported uncertainty about which GenAI tools were officially permitted.	Publish and regularly update an approved-tool list for administrative use.
Tool-approval workflows	Organizational legitimacy and procedural support	Participants hesitated to use GenAI when no formal approval pathway existed.	Establish a formal workflow for requesting, reviewing, and approving GenAI tools for departmental use.
Procurement arrangements and budget support	Resource availability	Access to paid tools or advanced functions was perceived as dependent on limited institutional resources.	Create procurement pathways and budget mechanisms for institutionally licensed GenAI tools.
Account and access rules	Technical access and equity	Uneven access to accounts, software, or networks limited staff adoption.	Provide standardized institutional accounts and equitable access rules across departments.
Training manuals and usage guidelines	Capacity building	Participants wanted clearer instructions on how to use GenAI appropriately in administrative tasks.	Develop practical training manuals, role-based examples, and standard operating guidance.
Privacy and provenance standards	Trust and verification	Staff expressed concern about privacy risks and how to verify whether AI-generated content was reliable and traceable.	Introduce privacy safeguards, provenance standards, and verification procedures for AI-assisted outputs.
Accountability procedures	Responsibility boundaries	Participants were unsure who would be responsible if AI-generated outputs caused errors.	Define accountability procedures for reviewing, approving, and using AI-assisted content in formal administrative work.

This table summarizes the main organizational and policy artifacts identified by participants and shows how they can be translated into actionable facilitating conditions for responsible GenAI adoption.

## Discussion

Grounded in the UTAUT [15], this study employed one-on-one semi-structured interviews to investigate the perceptions and attitudes of administrative staff in higher vocational colleges toward GenAI. It systematically analyzed the key factors influencing their acceptance of the technology. Within the participating colleges, the findings suggest that administrators generally maintain an open and positive attitude toward GenAI, particularly recognizing its value in enhancing work efficiency, supporting decision-making, and improving service quality. However, the adoption process appears to be heavily moderated by technological usability, organizational culture, social influence, and resource availability. Given the qualitative design, UTAUT moderators, such as age, administrative experience, prior AI exposure, department type, and institutional digital maturity, were interpreted descriptively rather than tested statistically. Accordingly, these findings offer analytically transferable insights rather than statistically generalizable claims.

Regarding performance expectancy, the findings confirm the influence of perceived usefulness as posited in Technology Acceptance Model (TAM) [16]. Administrators widely believe that GenAI contributes to improving work efficiency, streamlining administrative processes, and enhancing the quality of decisions. This aligns with recent studies on AI acceptance in university contexts and faculty GenAI use, where perceived improvement remains a central determinant of adoption [1,2,10]. However, unlike teaching-oriented studies that emphasize instructional preparation, feedback, or learning support, the present findings indicate that administrative staff primarily evaluate GenAI through its contribution to document production, data handling, procedural standardization, and service responsiveness. This suggests that for administrators, usefulness is inextricably linked to workflow reliability, institutional accountability, and service delivery rather than only instructional enhancement.

The results indicate that the learning threshold and operational complexity of GenAI are significant barriers to its adoption. Similar barriers have been reported in ChatGPT and professional-training contexts, where users’ confidence, AI literacy, and understanding of system limitations influence acceptance [8,19]. In the administrative context, effort is uniquely tied accountability risks. Errors in AI-assisted work do not just hinder personal learning, they potentially compromise compliance, service quality, or institutional credibility. Descriptively, participants with higher prior exposure to AI tools appeared to report fewer effort-related barriers and greater confidence in experimenting with GenAI, whereas participants with limited prior exposure more often emphasized unfamiliar terminology, uncertainty about correct operation, and the need for step-by-step training. These patterns should be interpreted as qualitative tendencies rather than statistically tested relationships. Compared with student- or faculty-focused ChatGPT adoption studies, effort expectancy in this study was less about personal learning convenience alone and more about whether staff could safely verify, revise, and take responsibility for AI-supported outputs in formal administrative procedures.

Regarding social influence, the research found that the organizational environment plays a critical role in guiding and shaping technology adoption among administrative personnel. Leadership advocacy, peer usage practices, a pro-innovation organizational culture, and formal institutional safeguards collectively constitute a social structure that influences individual behavior. This echoes existing research, which has shown that institutional endorsement, peer practices, and information quality can normalize AI use and reduce uncertainty [3,5,17]. For administrative staff, leadership signaling is paramount. It determines whether GenAI use is perceived as institutionally authorized or professionally risky. Notably, administrative experience appeared to shape how participants interpreted social influence: more experienced staff tended to emphasize formal leadership endorsement and institutional rules, while less experienced or technology-exposed staff more often referred to peer demonstration and informal knowledge sharing. In more risk-averse organizational environments, staff may hesitate to use GenAI without explicit authorization, even when they recognize its potential value.

With respect to facilitating conditions, the study highlights the foundational role of resource support systems in the effective promotion of GenAI. Structured training, adequate hardware infrastructure, stable network connectivity, approved access pathways, and performance-based incentives are all critical elements enabling administrative staff to engage with GenAI tools continuously and meaningfully. Recent studies on faculty GenAI use, professional AI training, and AI adoption ethics similarly emphasized the need for institutional support, training, and clear responsible-use guidance [9,10,19]. Institutional digital maturity further shaped participants’ perceptions of facilitating conditions. Staff from better-resourced settings tended to focus on practical application scenarios and workflow integration, whereas those from resource-constrained settings more frequently emphasized basic infrastructure, access to approved tools, training availability, and policy guidance. This cross-case pattern suggests that facilitating conditions may operate differently depending on institutional capacity. This cross-case pattern suggests that GenAI adoption is not a uniform process. Rather, resource disparities and procurement constraints create divergent adoption trajectories across the vocational education sector.

### Organizational and governance challenges

Beyond the four standard UTAUT constructs, the findings highlight organizational and governance challenges that uniquely constrain GenAI adoption. Administrative staff manage institutional documents, student information, personnel records, admissions procedures, finance-related processes, and cross-departmental coordination. These responsibilities render issues of data privacy, provenance, verification, and accountability far more salient than in individual pedagogical applications of GenAI.

Unlike students or faculty who may use GenAI for learning support or lesson preparation, administrative staff operate within formal workflows where errors directly impact institutional compliance, service delivery, institutional reputation, or resource allocation. Participants’ concerns about job security, output accuracy, and unclear responsibility boundaries indicate that adoption may be limited by administrative risk aversion as well as by technical barriers.

AI governance therefore emerged as a prerequisite for responsible adoption. Clear data-governance rules, approved-tool lists, tool-approval workflows, procurement procedures, privacy safeguards, and verification responsibilities are needed before GenAI can be embedded into formal administrative work. Without these safeguards, staff may perceive GenAI as useful yet remain hesitant to rely on it for tasks involving sensitive data or institutional accountability.

These findings align with recent AI adoption research emphasizing trust, ethical considerations, and responsible-use conditions [9], while demonstrating that these concerns are intensified in administrative settings. Crucially, the study suggests that organizational resistance should not be interpreted simply as individual reluctance. Instead, hesitation often reflects rational concerns regarding unclear authorization, uncertain responsibility boundaries, insufficient tool approval procedures, and the absence of institutional safeguards.

### Theoretical implications

This study offers an exploratory, context-specific application of the UTAUT framework within a non-Western administrative context. By shifting the focus from instructional settings to administrative personnel in Chinese higher vocational colleges, an understudied group in both domestic and international literature, the study demonstrates the framework’s adaptability to institutional management environments reshaped by AI. The findings enrich the social and organizational dimensions of UTAUT by suggesting three GenAI-specific contextual extensions.

First, policy clarity and governance readiness may extend facilitating conditions, because administrative staff evaluate not only whether resources are available but also whether GenAI use is formally authorized, regulated, and institutionally supported. Second, perceived usefulness is moderated by the extent to which staff believe AI outputs can be verified and responsibly used in professional use. Third, institutional capacity operates as a contextual moderator across the four UTAUT constructs, influencing how staff perceive usefulness, effort, social legitimacy, and support conditions.

Furthermore, ethical concerns regarding job security, data privacy, and accountability for AI-generated outputs, emerged as cross-cutting risk perceptions that are not fully captured by the original four UTAUT constructs. In administrative settings involving sensitive data, these concerns may weaken perceived usefulness and shape judgments of facilitating conditions [9]. While these insights are provisional due to the limited qualitative sample, they suggest that future UTAUT-based research should systematically examine prior AI exposure and institutional digital maturity through larger-scale or mixed-methods designs.

### Practical implications

The findings of this study offer several actionable insights for the effective integration of GenAI into vocational college administration.

Administrators’ recognition of GenAI’s potential to enhance workflow efficiency, improve decision quality, and personalize service delivery suggests that institutions should proactively identify and promote high-impact use cases, such as document automation, policy analysis, and internal communications, to demonstrate tangible value and build institutional momentum. These recommendations are most directly applicable to institutions with administrative conditions similar to the three participating colleges.

Capacity-building strategies must move beyond general technical skills to address cognitive overload and data governance. Training should be tailored to both AI exposure levels and specific administrative functions. Role-specific training is also needed. Finance staff may require guidance on data accuracy, compliance, and verification of AI-assisted reports; student affairs and admissions staff may need protocols for protecting student data and using GenAI in communication; academic affairs staff may benefit from examples related to scheduling, document processing, and policy retrieval; logistics staff may require practical support for integrating GenAI with existing equipment, procurement, and service-management workflows.

Furthermore, the influence of organizational and social factors points to the importance of leadership endorsement, peer collaboration, and cultural readiness. Institutions are advised to embed GenAI deployment within broader digital governance strategies that include pilot programs, feedback loops, recognition mechanisms, and staff participation in policy design. GenAI policies should be co-designed with professional-service staff rather than imposed top-down. Administrative personnel possess a unique understanding of operational risks, data flows, and approval procedures embedded in daily work. Practical verification strategies for AI-generated content and clear guidance on privacy, accountability, and appropriate administrative use should be included in these initiatives. Equally important, institutions should create safe experimentation spaces where staff can test GenAI tools without fear of blame for early-stage mistakes, provided that sensitive data and formal decisions are protected. Peer-sharing communities and safe reporting channels should also be established so that staff can exchange effective practices, report concerns, and identify implementation problems without reputational risk.

Finally, successful GenAI adoption in vocational administration requires systemic support at multiple levels. Beyond technical infrastructure and software availability, long-term sustainability depends on institutional policies that clarify accountability, promote inclusive access to AI resources, and establish clear ethical guidelines. For institutions with lower digital maturity, priority should be given to baseline infrastructure, approved access pathways, and practical support mechanisms before requiring widespread GenAI use. Such support should include clear procurement channels, data-protection requirements, provenance standards, and procedures for reviewing AI-assisted administrative outputs. Equity considerations are also important. Better-resourced colleges may benefit earlier from GenAI, while resource-constrained colleges may fall further behind if infrastructure, training, approved tools, and procurement support remain uneven. To reduce this risk, governance bodies could provide shared provincial training resources, minimum AI-infrastructure standards, approved-tool guidance, and inclusive access policies for colleges with weaker digital capacity.

## Conclusion

Grounded in the UTAUT framework, this study conducted an in-depth qualitative investigation of 16 administrative staff members from three representative higher vocational colleges in Henan Province. Through thematic analysis, the study identified that while the four core UTAUT constructs remain highly relevant, GenAI acceptance is uniquely shaped by policy ambiguity, governance readiness, perceived trustworthiness and controllability, institutional digital maturity, and ethical concerns. The findings provide insight into the behavioral logic of administrative personnel in the face of technological change and address an underexplored area concerning GenAI acceptance in the administrative domain of vocational education.

The results offer valuable implications for administrative practice in higher vocational institutions. Overall, administrative staff exhibited positive perceptions of GenAI, providing a basis for further exploration of AI-supported transformation in institutional governance. To effectively implement GenAI, institutions must move beyond technical deployment to focus on optimizing user experience, lowering adoption barriers, and establishing training systems and institutional safeguards. Institutions should also strive to foster a supportive organizational culture that encourages innovation, reinforces leadership-driven advocacy, and promotes a collaborative and participatory intelligent administrative ecosystem. Furthermore, integrating AI usage outcomes into performance evaluation and incentive mechanisms may encourage administrators’ engagement and foster a proactive, internally motivated approach to continued AI adoption.

### Limitations and future research

This study acknowledges several limitations that provide pathways for future inquiry.

In regards to the scope and generalizability, the sample consisted of 16 administrative staff from three higher vocational colleges in one Chinese province. While thematic saturation was achieved and institutional portraits support analytical transferability, the findings are not statistically generalizable to the broader vocational education sector.

Due to the qualitative design and small sample size, UTAUT moderators, such as age, gender, administrative experience, prior AI exposure, and institutional digital maturity, were interpreted descriptively rather than tested through regression, correlation, or structural modeling.

Although the study improved methodological transparency by reporting ATLAS.ti-assisted coding, a hybrid deductive–inductive strategy, inter-coder reliability, and a codebook, the analysis remains dependent on interview-based self-reports, which may not capture the full complexity of actual usage behavior.

Lastly, while the revised literature review integrated recent AI/ChatGPT acceptance studies and GenAI-specific issues such as trust, controllability, policy ambiguity, and ethical concerns, these proposed contextual extensions to UTAUT remain exploratory.

To build upon these findings, future studies should prioritize longitudinal tracking of behavioral change and comparative cross-institutional analysis to validate and refine the proposed GenAI-adapted UTAUT interpretation. Future research may also expand sample diversity by including vocational institutions across different regions, governance structures, and levels of digital maturity. Quantitative approaches such as path analysis or structural equation modeling could be used to examine the relationships among UTAUT constructs, GenAI-specific contextual factors, and adoption intentions. Finally, as GenAI technologies continue to evolve rapidly, further investigation is needed into algorithmic ethics, data governance, AI literacy training, and accountability mechanisms. Addressing these areas will be essential for constructing robust governance frameworks and supporting the responsible, standardized, and sustainable development of administrative systems in vocational education.

## Supporting information

S1 Table Participant characteristics. This table provides detailed participant characteristics, including institution, department, administrative role, years of experience, and prior AI exposure.
(DOCX)

S2 Table Finalized codebook for UTAUT-guided thematic analysis. This table links UTAUT dimensions with thematic codes, indicators, coding origins, and GenAI-specific emergent themes identified during analysis.
(DOCX)

S1 File Semi-structured interview guide. This file provides the final interview protocol used for the one-on-one interviews with administrative staff in higher vocational colleges.
(DOCX)
