# Supplementary material for: Exploring administrative staff’s acceptance of generative AI in Chinese vocational colleges: A UTAUT-guided thematic study
Source: PLoS One. 2026 Jul 17;21(7):e0346003. doi: 10.1371/journal.pone.0346003 (PMC13378991; doi:10.1371/journal.pone.0346003)
Supplement: S1 Table — This table provides detailed participant characteristics, including institution, department, administrative role, years of experience, and prior AI exposure. (DOCX) [file pone.0346003.s001.docx]

**S1 Table. Participant characteristics**

| Participant ID | Job Role (Department) | Years of Administrative Experience | Prior Exposure to AI Tools | Recruitment Channel |
| --- | --- | --- | --- | --- |
| P01 | Human Resources | 12 | Moderate (used translation/chat) | Department recommendation |
| P02 | Finance | 8 | Low (limited spreadsheet AI use) | Institutional email invitation |
| P03 | Student Affairs | 15 | None | Snowball sampling |

| P04 | Academic Affairs | 10 | High (strategic planning tools) | Department recommendation |
| --- | --- | --- | --- | --- |
| P05 | Leadership Office | 18 | Low | Direct nomination |
| P06 | Admissions & Employment | 6 | None | Department recommendation |
| P07 | Logistics | 9 | Low | Institutional email invitation |
| P08 | Academic Support Unit | 7 | Moderate | Snowball sampling |
| P09 | Student Affairs | 11 | Low | Department recommendation |
| P10 | Finance | 13 | Moderate | Institutional email invitation |
| P11 | Human Resources | 5 | None | Snowball sampling |
| P12 | Admissions & Employment | 4 | High | Department recommendation |
| P13 | Leadership Office | 16 | Moderate | Direct nomination |
| P14 | Academic Affairs | 14 | Low | Institutional email invitation |
| P15 | Logistics | 20 | None | Department recommendation |
| P16 | Academic Support Unit | 9 | High (strategic planning tools) | Snowball sampling |
