# Supplementary material for: Exploring administrative staff’s acceptance of generative AI in Chinese vocational colleges: A UTAUT-guided thematic study
Source: PLoS One. 2026 Jul 17;21(7):e0346003. doi: 10.1371/journal.pone.0346003 (PMC13378991; doi:10.1371/journal.pone.0346003)
Supplement: S2 Table — This table links UTAUT dimensions with thematic codes, indicators, coding origins, and GenAI-specific emergent themes identified during analysis. (DOCX) [file pone.0346003.s002.docx]

**S2 Table. Finalized codebook for UTAUT-guided thematic analysis**

| UTAUT Construct | Operational Definition (for this study) | Thematic Codes (examples) | Inclusion / Exclusion Rules | Example Indicators (data cues) | Coding Origin |
| --- | --- | --- | --- | --- | --- |
| Performance Expectancy | Perceived improvement in task performance, decision quality, service responsiveness due to GenAI | Document speed & quality; Decision support/analytics; Service personalization; Workflow standardization | Include statements about efficiency, accuracy, quality, responsiveness. Exclude training/resources (code under Facilitating Conditions) | “finish documents faster,” “better evidence for decisions,” “real-time replies” | Deductive |
| Effort Expectancy | Perceived ease/difficulty of learning and operating GenAI tools | Interface complexity; Learning burden; Error tolerance; Compatibility/usability | Include UI complexity, jargon, trial-and-error costs. Exclude lack of infrastructure (Facilitating Conditions) | “too many functions,” “hard to learn,” “fear of making mistakes” | Deductive |
| Social Influence | Influence of leaders, peers, norms, policies on intention/usage | Leadership endorsement; Peer modeling/sharing; Innovation culture | Include references to leaders/colleagues shaping usage; cultural climate. Exclude formal resources (Facilitating Conditions) | “director encouraged us,” “colleagues showed me,” “safe to experiment here” | Deductive |
| Facilitating Conditions | Availability of institutional supports enabling use | Training & help; Hardware/network; Access & security protocols; Incentive alignment | Include training, infrastructure, access controls, performance ties. Exclude personal dispositions (Effort/Performance) | “need structured training,” “PC too old,” “VPN/access path complicated” | Deductive |
| Ethical / Job-Security Concerns *(cross-cutting)* | Anticipated risks to roles, privacy, fairness, accountability | Job displacement anxiety; Data privacy/compliance; Accountability | Include explicit risk/ethics/privacy/job-loss concerns. Exclude generic difficulty (Effort) | “AI may take over tasks,” “data security worries,” “who is responsible?” | Inductive |
| Policy Ambiguity *(subsumed under Facilitating Conditions in reporting)* | Unclear rules about allowed use, boundaries, governance | Unclear usage boundaries; Approval uncertainty; Compliance ambiguity | Include uncertainty about policies; Map analytically to Facilitating Conditions when reporting | “not sure what’s allowed,” “no guideline yet,” “afraid of violating policy” | Inductive |
