# Supplementary material for: Exploring administrative staff’s acceptance of generative AI in Chinese vocational colleges: A UTAUT-guided thematic study
Source: PLoS One. 2026 Jul 17;21(7):e0346003. doi: 10.1371/journal.pone.0346003 (PMC13378991; doi:10.1371/journal.pone.0346003)
Supplement: S1 File — This file provides the final interview protocol used for the one-on-one interviews with administrative staff in higher vocational colleges. (DOCX) [file pone.0346003.s003.docx]

**S1 File. Semi-structured interview guide**

**Semi-Structured Interview Guide**

**Perceptions and Influencing Factors of Generative AI Acceptance among Administrative Staff in Higher Vocational Institutions**

**Section 1: Background Information**

1.1 Could you briefly describe your current role and responsibilities within the college?

1.2 How long have you been working in administrative positions in vocational education?

1.3 What prior experiences, if any, have you had with artificial intelligence tools (including GenAI)?

**Section 2: Perceptions and Attitudes toward GenAI**

2.1 When did you first hear about generative AI tools such as ChatGPT? From what sources?

2.2 What are your initial impressions of GenAI in general?

2.3 How do you personally feel about the idea of using GenAI in your daily administrative work?

**Section 3: Current Usage Practices**

3.1 Have you ever tried using GenAI tools in your work? If yes, in what ways (e.g., drafting documents, information retrieval)?

3.2 If not, what factors have prevented you from trying them?

**Section 4: Acceptance Factors (UTAUT Dimensions)**

4.1 Performance Expectancy: In what ways do you think GenAI could help you perform your tasks more effectively?

4.2 Effort Expectancy: How easy or difficult do you think it would be for you to learn and use GenAI tools?

4.3 Social Influence: How do the attitudes of your colleagues, supervisors, or institutional policies affect your willingness to use GenAI?

4.4 Facilitating Conditions: What kinds of support (training, infrastructure, policies) would you need to feel confident about using GenAI in your work?

**Section 5: Future Prospects and Concerns**

5.1 What opportunities do you see for GenAI to change vocational college administration in the next 3–5 years?

5.2 What concerns or risks (e.g., job security, data privacy, ethical issues) do you have about using GenAI?

5.3 If your institution were to formally introduce GenAI tools, what would help you adopt them most effectively?

**Section 6: Closing**

6.1 Is there anything else you would like to add about GenAI and its potential role in vocational administration?
